# A black phosphorus nanosheet-based RNA delivery system for prostate cancer therapy by increasing the expression level of tumor suppressor gene PTEN via CeRNA mechanism

**DOI:** 10.1186/s12951-024-02659-2

**Published:** 2024-07-04

**Authors:** Shunye Su, Leyi Liu, Qingfeng Fu, Minghao Ma, Na Yang, Ting Pan, Shengyong Geng, Xue-Feng Yu, Jianqiang Zhu

**Affiliations:** 1 Department of Urology, Tianjin Institute of Urology, The Second Hospital of Tianjin Medical University, Tianjin, 300211 China; 2 State Key Laboratory of Environmental Chemistry and Ecotoxicology, Research Center for Eco-Environmental Sciences, Chinese Academy of Sciences, Beijing, 100085 China; 3 Materials and Interfaces Center, Shenzhen Institutes of Advanced Technology, Chinese Academy of Sciences, Shenzhen, 518055 China; 4 Institute of Nanophotonics, College of Physics & Optoelectronic Engineering, Jinan University, Guangzhou, 511443 China

**Keywords:** Black phosphorus nanosheets, RNA delivery, ceRNA mechanism, PTEN, Prostate cancer

## Abstract

**Background:**

Prostate cancer (PCa) has a high incidence in men worldwide, and almost all PCa patients progress to the androgen-independent stage which lacks effective treatment measures. PTENP1, a long non-coding RNA, has been shown to suppress tumor growth through the rescuing of PTEN expression *via* a competitive endogenous RNA (ceRNA) mechanism. However, PTENP1 was limited to be applied in the treatment of PCa for the reason of rapid enzymatic degradation, poor intracellular uptake, and excessively long base sequence to be synthesized. Considering the unique advantages of artificial nanomaterials in drug loading and transport, black phosphorus (BP) nanosheet was employed as a gene-drug carrier in this study.

**Results:**

The sequence of PTENP1 was adopted as a template which was randomly divided into four segments with a length of about 1000 nucleotide bases to synthesize four different RNA fragments as gene drugs, and loaded onto polyethyleneimine (PEI)-modified BP nanosheets to construct BP-PEI@RNA delivery platforms. The RNAs could be effectively delivered into PC3 cells by BP-PEI nanosheets and elevating PTEN expression by competitive binding microRNAs (miRNAs) which target PTEN mRNA, ultimately exerting anti-tumor effects.

**Conclusions:**

Therefore, this study demonstrated that BP-PEI@RNAs is a promising gene therapeutic platform for PCa treatment.

**Graphical Abstract:**

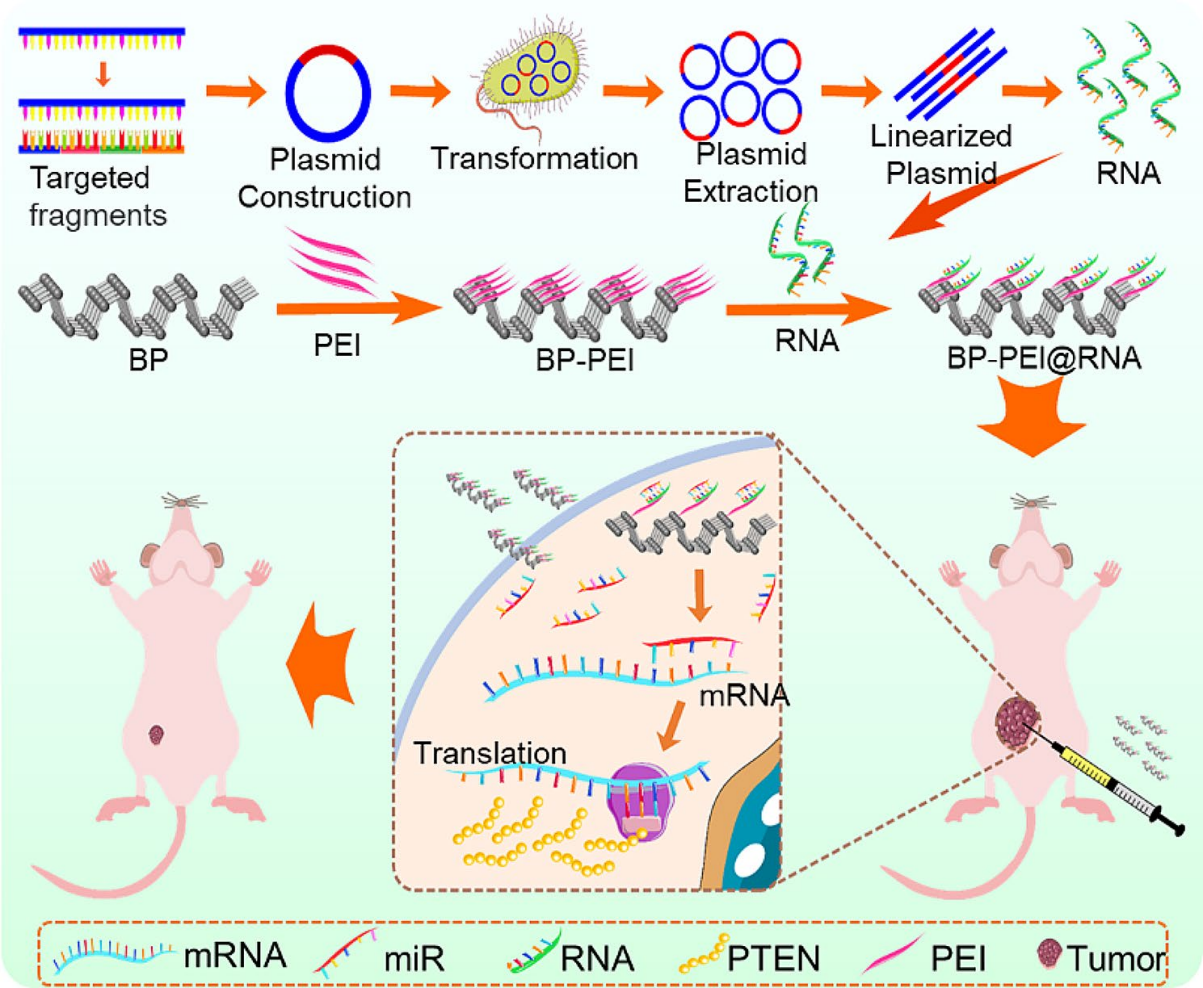

**Supplementary Information:**

The online version contains supplementary material available at 10.1186/s12951-024-02659-2.

## Introduction

Prostate cancer (PCa) is the second most common malignancy and is the fifth cause of cancer death in men worldwide [1]. In recent years, endocrine therapy, and radiotherapy along with surgical operation have contributed to a reduction in mortality, however, one-third of PCa patients will recur, advance to castration-resistant PCa stage, and develop distant organ metastases, which lack effective therapeutics and result in shorter overall survival [2]. Thus, there is a thirst for a novel and more effective strategy for PCa treatment currently.

Gene therapy strategies have demonstrated superior application potential to treat cancer diseases by altering the expression levels of target genes [3]. However, the low efficiency of cellular uptake, the low specificity of RNA release into the cytoplasm, and the rapid enzymatic hydrolysis of RNA in the abiotic internal environment are the major obstacles to the successful delivery of RNA to target cells [4]. In recent years, artificial nanomaterials have emerged as a promising vehicle for gene therapeutic agent delivery to overcome the inherent limitations of gene therapy [5]. And, it is demonstrated that nanomaterials as gene therapeutic agent carriers could deliver the gene drug cargo into cells by “Trojan Horse” effect, and possess the ability to reduce the degradation rate of gene therapeutic agents [6]. Over the past decades, nanomaterials with a variety of different chemical and physical properties, such as liposomes [7], carbon nanotubes [8], etc., have been used to construct drug delivery platforms, imaging and exhibit excellent therapeutic effects in cancer. However, many RNA vectors based on nanoplatforms face difficulties in achieving safe and rapid biodegradation. In addition, some nanomaterials are not essential elements of the human body, which inevitably generates immune responses and induces potential toxicity [9]. Compared with other nanomaterials, black phosphorus nanosheets (BP) have a higher surface-to-volume ratio due to the wrinkled lattice configuration, which could increase the drug loading capacity [10]. For instance, Yang et al. [11] designed a biocompatible biomimetic system for mRNA delivery using red blood-cell-derived nanoerythrocytes (NER) and BP, and the results showed that systematic delivery of coronavirus receptor-binding domain (RBD) mRNA significantly improved antibody titers and pseudovirus neutralization compared with NER without BP assistance. Therefore, BP could be used as a safe and effective delivery tool for RNA-based therapies. However, BP itself was also reported to be easily oxidized and degraded to nitrite ions and phosphate and naked BP with negative charge cannot efficiently load and deliver RNA [6, 11], which could be avoided by PEI modification since PEI is positively charged and could accomplish lysosomal escape through the “proton sponge effect” [12]. For example, Wang et al. used a survivin siRNA delivery system based on PEI-functionalized BP nanosheets to effectively inhibit tumor growth by silencing survivin expression [13]. Therefore, the unique properties of BP and the PEI modification that allows it to escape lysosomal lysis make BP-PEI a promising drug delivery system for cancer treatment. Here, we have designed a gene delivery system based on PEI-functionalized BP nanosheets (BP-PEI), which could efficiently load RNA, protect RNA from enzymatic degradation, and enhance intracellular RNA release.

PTEN is a typical tumor suppressor gene with PCa. Inactivation of PTEN due to genomic deletion or mutation is widely identified within primary prostate tumor samples and particularly within castration-resistant tumors [14]. Inhibition of PTEN expression results in activation of the PI3K-AKT pathway which plays a powerful role in promoting tumor progression, and cancer cells are ultrasensitive to even a subtle decrease in PTEN levels and activity, making PTEN an important therapeutic target for PCa therapy [15]. Recently, several studies have confirmed that long non-coding RNA PTENP1, the pseudogene of PTEN, which contains a highly homologous region upstream of the 3′UTR of PTEN could act as an “endogenous sponge” to share miRNA binding sites to block the degradation effect of miRNA on the PTEN mRNA by post-transcriptional control [16–18]. Thus, elevating the content of PTENP1 within the cells could improve the amount of PTEN proteins through the ceRNA mechanism.

To this end, we employed a two-dimensional biodegradable nanomaterial BP nanosheets with PEI modification to design a gene-drug nanocarrier, which showed excellent biocompatibility [10]. The gene drugs (RNA segments) were designed to increase the expression level of PTEN by the ceRNA mechanism and were synthesized according to the sequence of lncRNA PTENP1 as a template. To improve the loading efficiency of the negatively charged RNA segments, the surface of BP nanosheets was modified with positively charged PEI, so that the RNA segments could be loaded onto the BP-PEI nanosheets by electrostatic adsorption. We uncovered that BP-PEI could effectively load and transport RNA segments to the interior of the cell, meanwhile, protecting RNA segments from enzymatic degradation. Finally, BP-PEI@RNAs inhibited prostate tumor progression by improving the expression level of the tumor suppressor gene PTEN. Taken together, our results revealed the potential of a BP-based RNA delivery system that could be a promising method for gene therapy in the treatment of PCa.

## Materials and methods

### Synthesis RNAs

The total mRNA of RWPE-1 cells (human normal prostate epithelial cell) was extracted by Trizol method and reverse transcribed into cDNA, and all the steps and PCR setup were performed according to the guide of Fast-Quant RT Kit (TianGen, China) to obtain cDNA. Then, the cDNA was used as a template for the PCR to synthesize target RNAs. The sequence of the PTENP1 gene (GeneID:11,191) was sequentially divided into four segments as templates to synthesize four RNAs (RNA1, RNA2, RNA3, RNA4) by PCR method, and the primers were summarized in the supplementary materials (Table S1). Then, the RNAs were gel purified with the Wizard SV Gel and PCR Clean-Up System (Promega Corporation, USA), and stored at -80 ℃.

### Construction, transformation, amplification and extraction of the plasmid

Four RNAs were inserted into pGEM-T Easy Vector System I (Promega, USA) to synthesize plasmids, respectively. Then, 2 µg plasmid was mixed with 100 µL of competent Escherichia coli and an ice bath was performed for 30 min. It was placed in a 42 ℃ water bath with thermal shock for 90 s and immediately placed in an ice bath for 2 min. And 900 µL Luria-Bertani (LB) liquid medium was added, which was prepared by adding 10 g tryptone (OXOID, UK), 10 g NaCl (Solarbio, China), 5 g yeast extract (OXOID, UK), deionized water to 1000 mL, and then autoclaved and added 10 mg ampicillin. Then the mixture was stirred at 225 rpm at 37 ℃ for 1 h, after which the bacterial solution was planted on an agar plate containing 100 µg/mL ampicillin, which was then inverted and kept at 37 ℃ overnight. After colonization, bacterial colonies of approximately 1 mm were selected with a sterile ring in 25 mL LB liquid medium containing 100 µg/mL ampicillin and shaken at 180 rpm at 37 ℃ for 16 h. Part of the bacterial solution obtained was used for sequencing to verify the sequence. The rest was centrifuged at 4 ℃ at 6000 rpm for 10 min. After discarding the supernatant, the QIAGEN Plasmid Midi Kit (QIAGEN, Germany) was used to extract the plasmids in the bacterial solution.

### Linearization, purification and recovery of the plasmid

A 50 µL plasmid linearization system was used for plasmid linearization. Specifically, 5 µL NcoI-HF (New England Biolabs, USA), 5 µL CutSmart Buffer (New England Biolabs, USA) and 10 µg plasmid were added into the enzyme-free EP tube, and enzyme-free water was added to the system to 50 µL. After full mixing, the linearized plasmid was obtained after incubation at 37 ℃ for 6 h. The linearized plasmid was gel purified with the Wizard SV Gel and PCR Clean-Up System (Promega Corporation, USA), and stored at -20 ℃.

### Synthesis of RNA by in vitro transcription

Linearized DNA was synthesized into RNA through in vitro transcription. Specifically, 10 µL of 5× transcription buffer (Thermo Fisher Scientific), 10 mM of ATP/GTP/CTP/UTP (Thermo Fisher Scientific) 1 µL each, 1 µg of linearized DNA, 1.25 µL RNAase inhibitor (Thermo Fisher Scientific), 1.5 µL T7 polymerase (Thermo Fisher Scientific), DEPC water to 50 µL were added into the enzyme-free EP tube; then, the system was incubated at 37 ℃ for 2 h, and RNA was transcribed and then stored at -20 ℃ for later use.

### Construction of BP-PEI@RNAs

BP nanosheets were prepared by the liquid exfoliation method described in a previous study [19], centrifuged at 4 ℃ (16,000 rpm, 60 min), and washed three times with enzyme-free water to obtain a well-dispersed BP aqueous dispersion solution in a concentration of 1 µg/ml. Polyetherimide (PEI) (2.5k, 10 mg/mL, CAS: 9002-98-6, Sigma-Aldrich) was then added to the solution at a volume ratio of 1:1.5 for BP: PEI, and then the mixture was sonicated in the ice water bath for 3–4 h to obtain BP-PEI. BP-PEI (1 µg/ml) was resuspended and incubated with RNA (0.1 µg/ml) (BP to RNA mass ratio of 1:0.4) at 4 °C for 10 min with continuous rotational mixing to obtain BP-PEI@RNAs platform.

### Characterization of BP@RNA

The diameter and surface characteristics of BP nanosheets, BP-PEI and BP-PEI@RNAs were characterized by transmission electron microscope (TEM, JEOL JEM-2100 F, Japan) and atomic force microscope (AFM, Dimension Icon, Germany). Raman spectroscopy analysis of them was performed using a Raman spectrometer (Horiba scientific LabRAM HR evolution). The hydrated particle size and zeta potential were analyzed by nanoparticle size analyzer (Zetasizer Nano ZS90, Malvern, UK).

### Entrapment efficiency (EE%)

RNA EE% in BP-PEI was determined as previously described [20]. Briefly, RNA-loaded BP-PEI was centrifuged at 40,000 ×g for 20 min, and then the amount of uncaptured RNA in the supernatant was measured using RiboGreen Assay. EE% was calculated by the following formula:

$$\eqalign{{\rm{RNAEE}}\% & {\rm{ = }} \cr {{{\rm{ Amount of total RNA - Amount of uncaptured RNA }}} \over {{\rm{ Amount of total RNA }}}} \times {\rm{100}} \cr}$$

### Evaluation of RNA stability

The ability of the BP-PEI@RNA complex to protect RNA against RNase A digestion was investigated by agarose gel electrophoresis. Briefly, the optimal BP-PEI@RNA1 formulation (BP-PEI: RNA = 1:0.4) containing 1 µg RNA was incubated with RNase (0.01 µg/mL) (Sigma, USA) in PBS (pH 7.4) for 0, 15, 30 and 60 min at 37 °C. RNA was transferred from BP-PEI by incubating the samples with 1 µL of 1 mg/ mL sodium dodecyl sulfate (SDS) solution (25 °C, 5 min). The same amount of RNase-treated free RNA was used as a control. The extracted RNA was subjected to 1% agarose gel electrophoresis at 120 V for 10 min. The resulting gels were stained with SYBR Green (Invitrogen, USA) and visualized on an imaging system.

### RNA release profile

RNA release of the BP-PEI@RNA complex was assessed at various pH conditions (3, 5, 7, 9, and 11) and at various protein concentrations (2.5%, 5%, 7.5%, and 10% FBS). For solutions with different pH values, the pH was adjusted with HCl or NaOH using a pH meter (Biofrontier Technology). For solutions with different protein concentrations, a certain volume of FBS was added to RPMI 1640 medium. The BP-PEI@RNA solution was incubated at 37 ℃ and 100 rpm in a shaker. At each time point (1, 2, 4, 6, 12, 24, 48 and 96 h), the supernatant was collected by centrifugation at 15,000 rpm for 30 min, and the amount released was determined.

### Fluorescent labeling of RNA

In the process of in vitro transcribed RNA preparation, 6-FAM or cy3 labeled ribonucleotides were used as fluorescent substrates instead of non-fluorescent labeled ribonucleotides, and RNA was synthesized according to the above process “Synthesis of RNA by in vitro Transcription” to obtain 6-FAM or Cy3 labeled RNA.

### Uptake and subcellular distribution of BP@RNA

All PC3 cells were cultured in RPMI 1640 medium (Gibco BRL Life Technologies Inc., Waltham, MA, USA) containing 10% FBS (Gibco BRL Life Technologies Inc.) and 1% antibiotics (Gibco BRL Life Technologies Inc.) and incubated at 37 ◦C in a humidified incubator with 5% CO_2_. When PC3 cells reached 50-60% confluence, they were exposed to the 6-FAM- and Cy3-labeled BP-PEI@RNA (1 µg/mL BP; 0.4 µg/mL RNA; BP-PEI@RNA: The mass ratio of BP-PEI and RNA was 1:0.4), respectively. The cellular uptake of BP-PEI@RNA-6FAM in PC3 cells was observed by CLSM at different time points (1, 3, and 6 h post exposure). The subcellular localization of PEI@RNA-Cy3 in PC3 cells was detected by co-staining with lysosomes and observed by CLSM at different time points (6, and 24 h post exposure).

### Live/ dead staining

When the confluence reached 50-60%, PC3 cells were exposed to BP-PEI@RNA (1 µg/mL BP; 0.4 µg/mL RNA; The mass ratio of BP-PEI and RNA was 1:0.4), and the cells were then stained with the Viability/Cytotoxicity Assay Kit for Animal Live & Dead Cells (Proteintech, China) at 24 h post exposure. The fluorescence signal of the cells was observed by CLSM.

### Cell viability test

PC3 cells were cultured in 96 well plates, when the confluence reached 50-60%, these cells were exposed to the free RNAs (0.4 µg/mL) or BP-PEI@RNA (1 µg/mL) for 24 h, and then the cell viability was tested by Cell Counting Kit-8 assay (MedChemExpress, USA).

### Autophagy, apoptosis and cell cycle test

PC3 cells were cultured in RPMI 1640 medium (supplemented with 10% fetal bovine serum and 1% penicillin/streptomycin). When 50-60% confluence was reached, these cells were exposed to BP-PEI@RNA (1 µg/mL BP; 0.4 µg/mL RNA) for 24 h. PC3 cells were harvested and stained with Lyso Tracker Green DND-26 (Cell Signaling Technology, USA), Annexin V-FITC apoptosis assay kit (Beyotime Biotechnology, China) and Cell Cycle Staining Kit (Abbkine, China).

### RT-qPCR

PC3 cells incubated with BP-PEI@RNA for 24 h were harvested. The miRNA of PC3 cells was extracted according to the guide of FastQuant RT Kit (With gDNase) (TianGen, China), and then reversely transcribed into cDNA *in vitro.* RT-qPCR was performed using Universal SYBR Green qPCR Supermix (US EVERBRIGHT, China). U6 was used as an internal control. The expression of miRNAs was quantified by 2^−ΔΔCT^ method. The primers were summarized in Table S2.

### Western-blot

PC3 cells were harvested and washed 3 times with RPMI 1640 medium. Then, these cells were lysed by RIPA lysis buffer (Pierce IP Lysis Buffer, USA) and the protease inhibitor cocktail was added to inhibit the degradation of total proteins. Protein concentration was detected by BCA protein assay kit (Solarbio Science & Technology Co., Ltd., Beijing, China). Equal proteins were used for sodium dodecyl sulfate-polyacrylamide gel and then incubated with different primary antibodies (anti-β-actin, anti-GAPDH, anti-PTEN, anti-P-PI3K, anti-PI3K, anti-P-AKT, anti-AKT, anti-LC3-I, anti-LC3-II, anti-p62, anti-cleaved caspase 3, anti-cleaved caspase 8, anti-cleaved caspase 9, anti-Bax and anti-Bcl-2) (Proteintech, China) at 4 ℃ overnight and then incubated with secondary antibodies at room temperature for 1 h. Finally, the expression of different proteins was visualized by BIO-RAD ChemiDoc XRS chemiluminescence system (Bio-Rad Inc., CA, USA).

### Animal experiment

With the approval of the Institutional Animal Care and Use Committee of Yi Shengyuan Gene Technology (Tianjin) Co., Ltd., male Balb/C nude mice at the age of 4 weeks were purchased from HFK Co. (Beijing, China). We constructed the PC3 subcutaneous tumor model by inoculating the collected PC3 cells (2 × 10^6^) with Matrigel in an equal volume ratio into the left abdominal subcutis of each mouse. The therapeutic intervention was performed 2 weeks after model establishment, and equal volumes of PBS (15 µl), BP (20 µg), RNA2 (12 µg) and BP (20 µg)-RNA2 (12 µg) were treated via intratumoral injection at 4 days intervals for a total of 4 times, respectively. The whole process of material preparation and injection followed the sterile principle. The length and diameter of the tumor were measured with vernier calipers and then the tumor growth curve was calculated and plotted according to the formula. On the 35th day, the mice were sacrificed and the tumors were harvested for weight measurement and histological examination.

### Tunel staining

Tumor tissues were sectioned and stained with CoraLit-594 TUNEL Assay Apoptosis Detection Kit (Proteintech, China) for cellular apoptosis detection. All the steps were performed under the guidance of the kit and the cells undergoing apoptosis were observed by CLSM.

### Immunohistochemistry staining

Paraplast-fixed tumor tissues were sectioned, deparaffinized, dehydrated, and antigen retrieved in citrate buffer (pH = 6.0). The sections were then incubated with different primary antibodies (anti-PTEN, anti-Ki67, anti-p62, anti-caspase 8, and anti-Bcl-2) (Proteintech, China) at 4 ℃ overnight. The sections were then incubated with goat anti-rabbit IgG H&L coupled to horseradish peroxidase (HRP; Abcam) for 30 min at room temperature, and protein expression was visualized by 3-3-diaminobenzidine (DAB) staining and observed under an optic microscope.

### Statistical analysis

Statistical analysis was performed using GraphPad Prism 10 software and presented as mean ± SD. Comparisons were performed using an independent t-test or one-way ANOVA test and differences between groups were considered statistically significant at *P* < 0.05.

## Results and discussion

### BP-PEI@RNA construction and physicochemical characterization

**Fig. 1:**
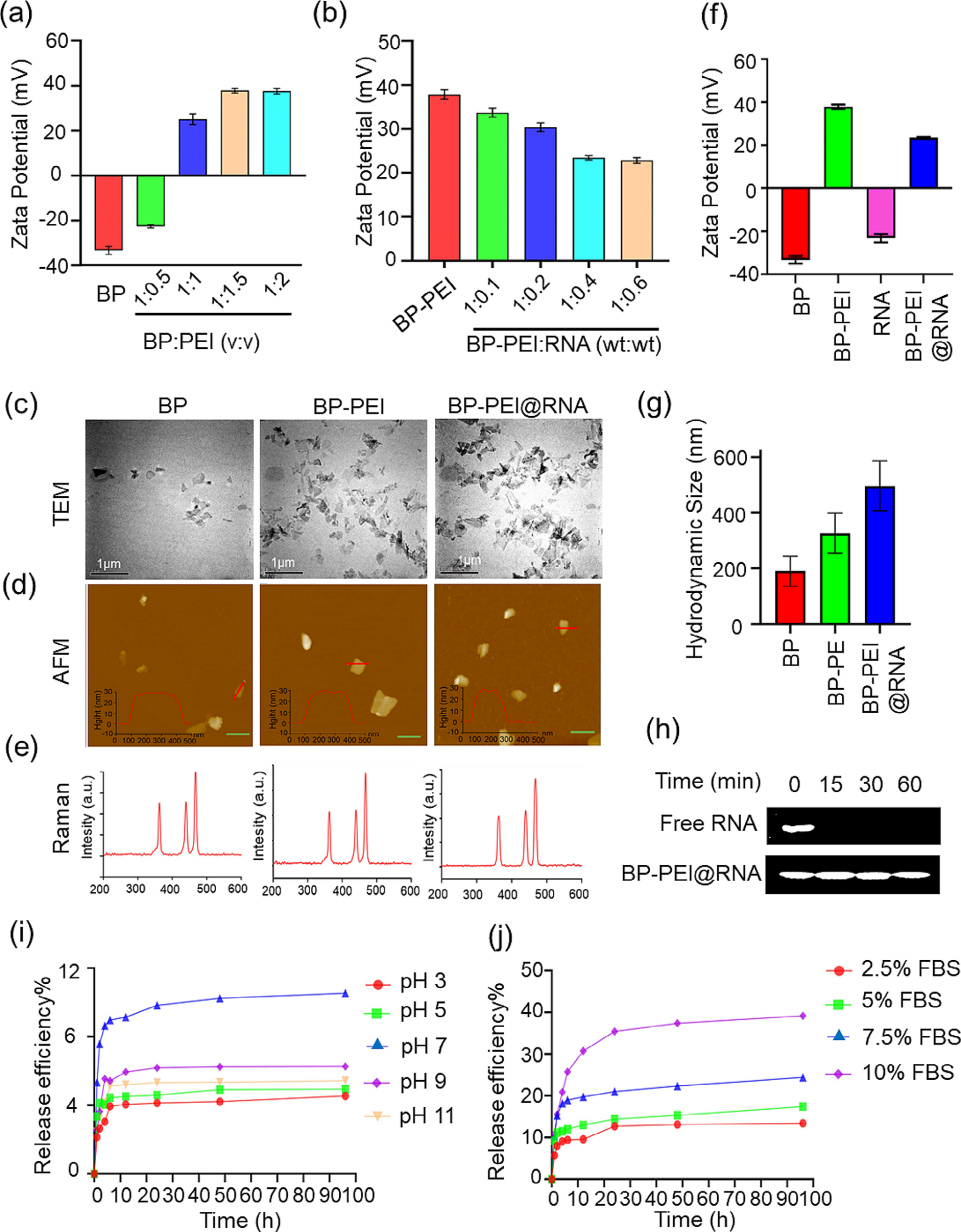
(**a**) Zeta potential of BP-PEI complexes in different volume ratios of BP: PEI. (**b**) Zeta potential of BP-PEI@RNA complexes in different weight ratios of BP-PEI: RNA. Representative TEM (scale bar = 1 μm) (**c**), AFM (scale bar = 500 nm) (**d**) and Raman spectral (**e**) of BP, BP-PEI and BP-PEI@RNA. The zeta potential (**f**) and the hydrodynamic particle size(**g**) of BP, BP-PEI and BP-PEI@RNA. (**h**) Stability of RNA against RNase before and after being adsorbed onto BP-PEI. Free RNA and BP-PEI@RNA (BP-PEI: RNA = 1:0.4) were incubated with RNase (0.01 mg/mL), and the RNA was analyzed by agarose gel electrophoresis at different incubation time points. In vitro release profile of RNA from BP-PEI@RNA complexes under various pH conditions (**i**) and in RPMI 1640 with different FBS concentrations (j)

The BP nanosheets adopted in this study were prepared as previously study reported [21]. Besides, it has been shown that BP nanosheets are negatively charged, so they cannot effectively load and deliver RNA that is also negatively charged [13]. Additionally, PEI could achieve lysosomal escape through the “proton sponge effect” to avoid the degradation of the nanocomposites by lysosomal enzymes [12]. Therefore, PEI was pre-modified on the surface of BP nanosheets before RNAs loading. To determine the optimal volume ratio of BP to PEI, we measured the zeta potential of BP-PEI with different volume ratios, as shown in Fig. 1a. The results showed that with the increase of PEI, the zeta potential of BP-PEI changed from negative to positive, and then became stable when the ratio of BP to PEI was 1:1.5, which indicated that the potential and stability of BP-PEI would not increase significantly even if the amount of PEI were increased. Therefore, a volume ratio of BP to PEI of 1:1.5 would be selected for the following experiments. Considering that the sequence of PTENP1 is too long to be high fidelity synthesized in vitro, thus, the whole length of PTENP1 sequence was sequentially divided into four segments with a length of about 1000 nucleotide bases to synthesize four different RNA fragments as gene drugs (RNA1, RNA2, RNA3, RNA4; Fig. S1). Four RNA fragments were loaded on the BP-PEI, respectively, and BP-PEI@RNA2 was randomly chosen as a representative to illustrate the physicochemical properties and subcellular localization of BP-PEI@RNA. Similarly, to determine the optimal mass ratio of BP-PEI to RNA, we measured the zeta potential of BP-PEI@RNA with different mass ratios, as shown in Fig. 1b. It has been showed that the zeta potential of BP-PEI@RNA was stable when the mass ratio of BP-PEI to RNA was 1:0.4, which indicated that the potential and stability of BP-PEI@RNA would not change significantly even if the amount of BP-PEI were increased. In terms of RNA entrapment, as shown in Fig. S2, the encapsulation efficiency of BP-PEI decreased from 100% when the mass ratio was 1:0.1 to 99.7% when the mass ratio reached 1:0.4 and to 92.0% when the mass ratio reached 1:0.6 with the increase of RNA under the same BP-PEI. Therefore, a mass ratio of BP-PEI to RNA of 1:0.4 was selected for the following experiments. The physicochemical characterization data of BP, BP-PEI and BP-PEI@RNA are shown in Fig. 1a-f. BP nanosheets were ultrathin nanosheet layers under observation by transmission electron microscopy (TEM) and it seemed that PEI modification and RNA loading did not change the morphology of BP nanosheets (Fig. 1c). The atomic force microscopy (AFM) showed the average lateral size of BP, BP-PEI and BP-PEI@RNA mainly distributed in the range of 100–400 nm, with a thickness of about 20–30 nm (Figs. 1d and S3). The typical Raman spectral data manifested the same representative band of BP, BP-PEI and BP-PEI@RNA (~ 360.8 cm^− 1^, ~ 438.7 cm^− 1^ and ~ 465.8 cm^− 1^) as shown in Fig. 1e. BP nanosheets and RNA were negatively charged with an average zeta potential of about − 33.33 ± 1.72 mV and − 23.20 ± 1.92 mV, and presented a positive surface charge of 37.83 ± 1.07 mV and 23.43 ± 0.55 mV post PEI modification and RNA loading, respectively (Fig. 1f), which was generally considered that the BP-PEI@RNA had sufficient mutual repulsion to form a stable aqueous dispersion according to the previous report [22]. The hydrodynamic particle size of BP nanosheets was around 200 nm, and it increased to around 300 nm and 500 nm post modified with PEI and further loaded with RNA, respectively (Fig. 1g). Gel electrophoresis images showed that RNA stripes in the BP-PEI@RNA group remained largely uniform after RNase treatment, whereas free RNA was almost completely degraded within 15 min Fig. 1h, indicating that the BP-PEI@RNA complex effectively protected RNA from nuclease degradation.

Following the successful formation of the BP-PEI@RNA complex, the effects of acidity/alkalinity (pH) and protein on its RNA release were investigated. The release profiles of BP-PEI@RNA were evaluated at pH values of 3, 5, 7, 9 and 11, and at different protein concentrations (RPMI 1640 containing 2.5%, 5%, 7.5%, and 10% FBS). RNA bound strongly to BP-PEI, and the escape rates of RNA from the complex were low at both alkaline and acidic conditions, while about 10.5% of RNA was released from the BP-PEI@RNA complex at neutral pH value (Fig. 1i). To better mimic the physiological environment, we also investigated the effect of proteins on RNA release. Interestingly, the amount of RNA released was elevated in the presence of FBS, as in RPMI 1640 containing 10% FBS, 39.2% of RNA was consistently released from the complex within 96 h (Fig. 1j). It has been reported that negatively charged proteins could competitively bind nanomaterials and enhance drug release, and there are various biological polyanions that competitively displace nucleic acids on nanoparticles in intracellular environments [23], which may lead to increased RNA release from BP-PEI@RNA in intracellular environments.

### BP nanosheets could efficiently transport rna segments into pc3 cells and further into the lysosomes

**Fig. 2:**
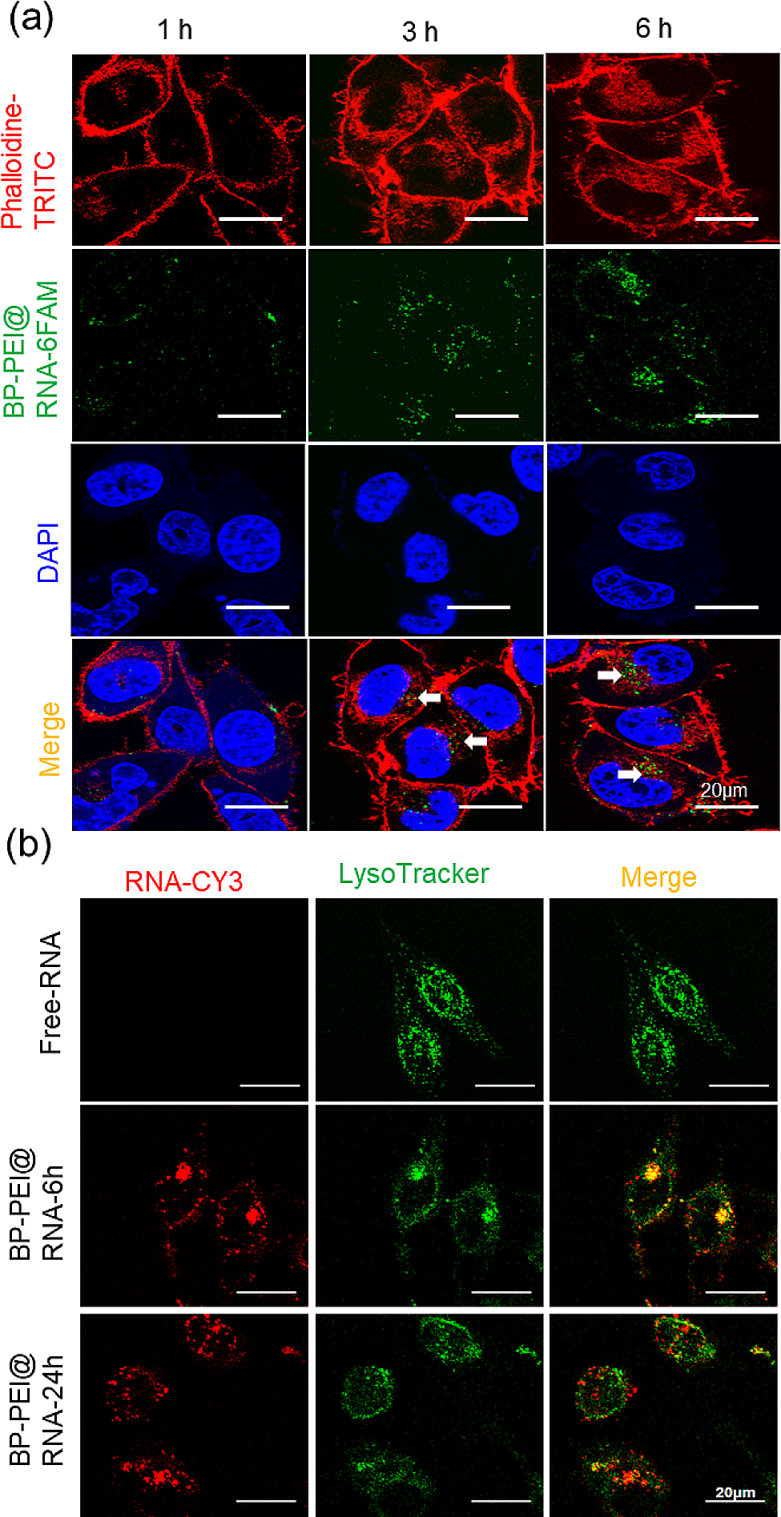
Cellular uptake and subcellular distribution of BP-PEI@RNA. (**a**) The cellular uptake of BP-PEI@RNA. PC3 cells were treated with BP-PEI@RNA-6FAM (in green) for 1, 3, and 6 h, then the nucleus was stained by DAPI (in blue) and the cytoskeletal structure was stained by Phalloidine-TRITC (in red), and the quantitative data of the fluorescence (in purple), scar bar = 20 μm; (**b**) Subcellular distribution of BP-PEI@RNA. RNA-Cy3 and BP-PEI@RNA-Cy3 (both in red) were employed to treat PC3 cells for 6 and 24 h, and the cells were then stained by LysoTracker to track the lysosomes (in green). The subcellular distribution of BP-PEI@RNA was visualized by confocal laser microscopy, scar bar = 20 μm

To verify whether BP nanosheets could transport RNA segments to the cell interior, confocal laser scanning microscopy (CLSM) analysis was performed after PC3 cells were treated with BP-PEI@RNA2 for 1, 3 and 6 h. The fluorescent molecular probe 6-carboxyfluorescein (6-FAM) was adopted to label nucleotides (in green) when RNA was synthesized. As shown in Fig. 2a, CLSM images revealed a mass of RNA entering into the cytoplasm (indicated by white arrows) at 3 h post BP-PEI@RNA treatment, and much more RNA accumulated in the cytoplasm at 6 h post BP-PEI@RNA treatment. Thus, we confirmed that BP-PEI nanosheets could effectively transport RNA into the interior of the cell. To further visualize the location of BP-PEI@RNA after it entered into the cell, co-staining of lysosomes and RNA was performed. RNA was labeled with sulfo-cyanine3 (Cy3) in red and lysosomes were stained in green using Lyso-Tracker. As shown in Fig. 2b, a mass of red fluorescence was observed which indicated as BP-PEI@RNA at 6 h and 24 h post BP-PEI@RNA treatment. The red fluorescence fused with green fluorescence showed yellow fluorescence at 6 h post BP-PEI@RNA treatment, indicating that the BP-PEI@RNA that entered the cells at this time was located in lysosomes. However, at 24 h after BP-PEI@RNA treatment, red fluorescence appeared in the cytoplasm. We speculated that some RNA was released into the cytoplasm, which might be considered due to the acid sensitivity of PEI-functionalized BP nanosheets [13]. In addition, a part of the yellow fluorescence signal could still be observed at 24 h after BP-PEI@RNA treatment, indicating that some BP-PEI@RNA cannot escape from lysosomes. Additionally, there is no red fluorescence signal observed with RNA-CY3 alone treatment cells, demonstrating that the RNA segment could not get into the interior of the cells, which was due to the large molecular weight and strong negative charge of RNA, which made it difficult for them to enter the cell [24]. Afterwards, we endeavored to address the resultant influence of BP-PEI@RNA on the proliferation, autophagy and apoptosis of PC3 cells in vitro and in vivo.

### BP-PEI@RNAs treatment inhibited the proliferation and viability of pc3 cells by elevating the expression level of PTEN

**Fig. 3:**
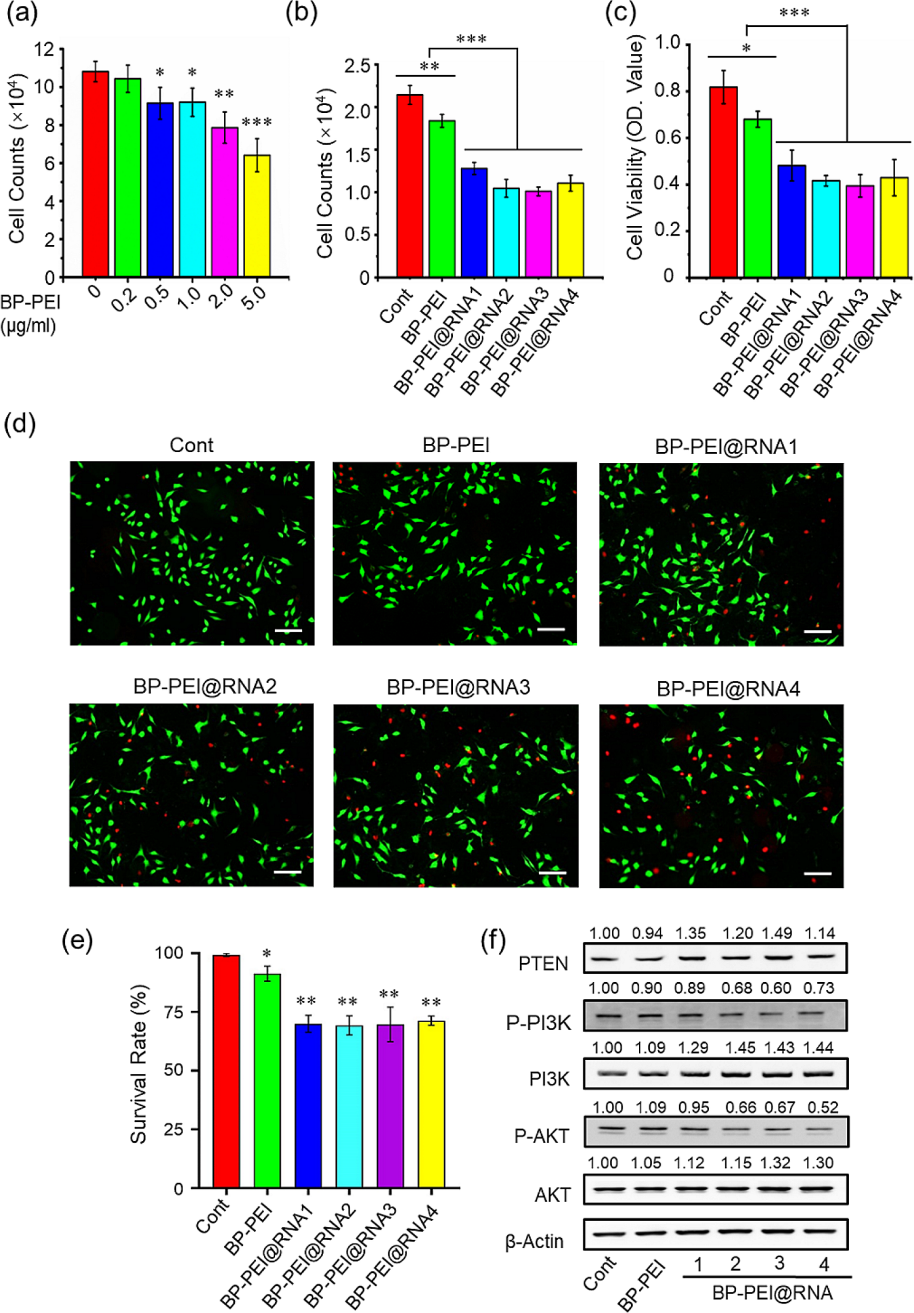
Effects of BP-PEI@RNAs treatment on the cellular viability and proliferation of PC3 cells in vitro. (**a**) PC3 cells were treated with BP-PEI at various concentrations (0, 0.2, 0.5, 1.0, 2.0, and 5.0 µg/mL) for 24 h, and the cell counting assay was conducted to assess the cytotoxicity of BP-PEI per se on PC3 cells; Cell counting assay (**b**), CCK8 assay (**c**) and cell live/ dead staining (**d-e**) were then adopted to determine the effect of BP-PEI@RNAs on proliferative capacity and viability of PC3 cells. (**f**) The protein expression levels of PTEN, P-PI3K, PI3K, P-AKT, and AKT were determined by western-blot assay post PC3 cells were treated with BP-PEI@RNAs for 24 h. * indicated *P* < 0.05, ** indicated *P* < 0.01, *** indicated *P* < 0.001

Firstly, the cytotoxicity of BP-PEI was determined by cell count analysis at the concentrations of 0.2, 0.5, 1.0, 2.0 and 5.0 µg/mL (Fig. 3a), and it showed a positive correlation with the treatment dose. Finally, we here selected 1.0 µg/mL as the treatment dose of BP-PEI in the following experiments. Next, four different pieces of RNAs (RNA1, RNA2, RNA3 and RNA4) were loaded onto BP-PEI at the mass ratio of 1:0.4, respectively. Cell counting analysis, Cell Counting Kit-8 analysis, and cell dead/living staining were adopted to evaluate the inhibition effects of BP-PEI@RNAs on the cellular viability and death of PC3 cells. The data showed that four pieces of BP-PEI@RNAs were all able to inhibit the proliferation of PC3 cells and induce cell death, and there was no significant difference among them (Fig. 3b-e). Meanwhile, the cellular viability of PC3 cells exposed to free RNA (four-segment RNA alone and in combination) was also evaluated and the results suggested that free RNA treatment had no effect on the cellular viability of PC3 cells (Fig. S4), probably the reason that free RNAs could not enter into cells by itself and easy to be degraded. Furthermore, western blot analysis was adopted to determine the expression level of the target protein PTEN and its downstream effector molecules (PI3K and AKT) which play a crucial role in cellular proliferation. We found that four BP-PEI@RNAs treatments were all able to elevate the expression level of PTEN, and decreased the expression level of P-PI3K and P-AKT which were the activated state of PI3K and AKT (Fig. 3f). These data suggested that BP-PEI@RNAs established in this study have an obvious inhibitory effect on the proliferation of PC3 cells by increasing the expression level of tumor suppressor gene PTEN, and four different BP-PEI@RNAs showed almost equal efficiency.

### BP-PEI@RNAs treatment induced cell cycle arrest of PC3 cells

**Fig. 4:**
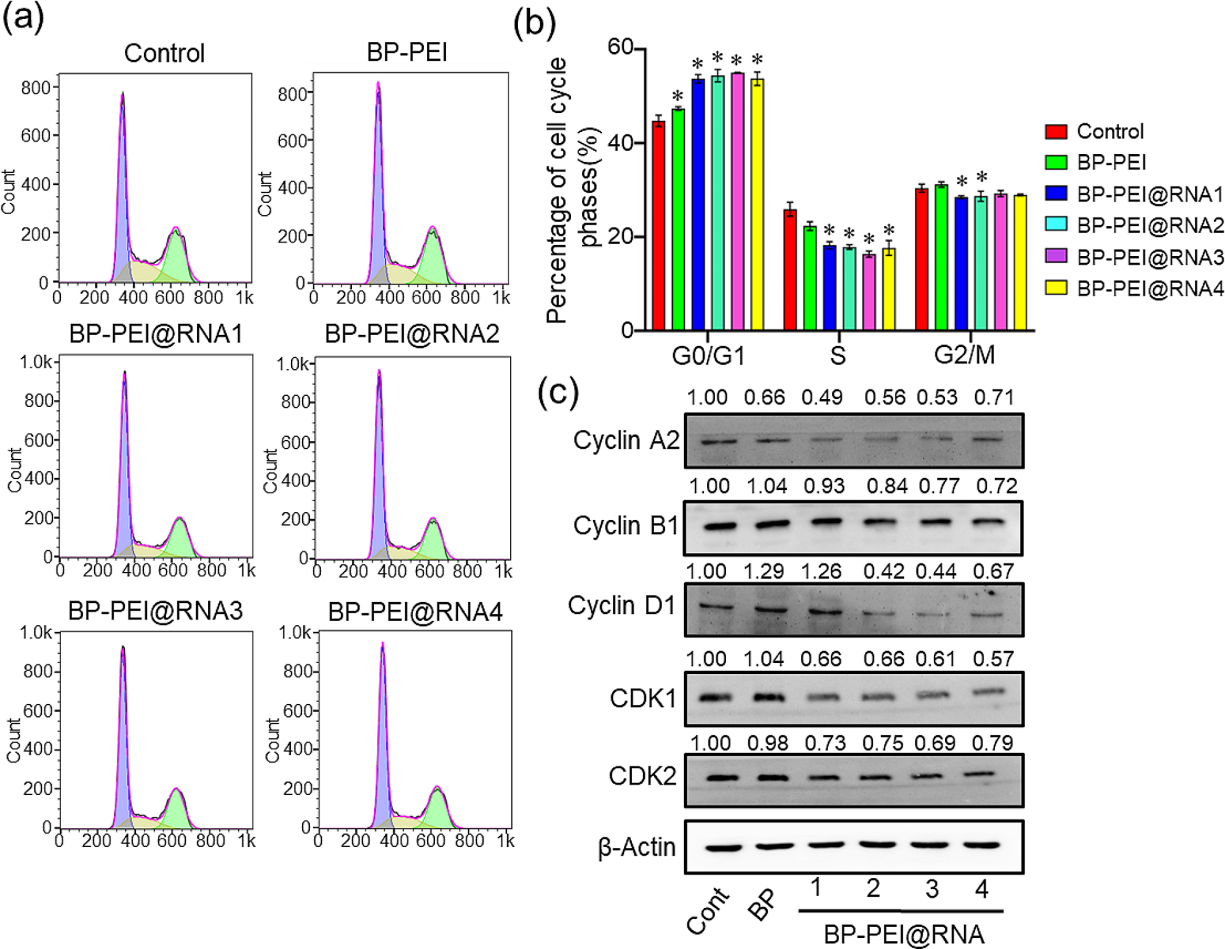
The effect of BP-PEI@RNAs treatment on cell cycle of PC3 cells. PC3 cells were treated with BP-PEI@RNAs for 24 h and stained with Cell Cycle Staining Kit for cell cycle analysis (**a**), and the quantified data was presented in (**b**). (**c**)The total proteins of PC3 cells were extracted post BP-PEI@RNAs treatment and the Western-Blot assay was employed to evaluate the expression level of cell cycle checkpoint proteins Cyclin A2, Cyclin B1, Cyclin D1, CDK1 and CDK2. * indicated *P*<0.05

Since PTEN is involved in the regulation of cell cycle progression which is the origin of rapid proliferation of cancer cells [25], increasing the expression of PTEN to induce cell cycle arrest is considered an effective anti-tumor method. Here, cell cycle analysis was performed on PC3 cells treated with BP-PEI@RNAs. As shown in Fig. 4a-b, exposure of PC3 cells to four BP-PEI@RNAs respectively resulted in an increasing number of cells distributed in the G0/G1 phase and fewer cells distributed in the S and G2/M phases. Thus, cell cycle analysis demonstrated that BP-PEI@RNAs treatment effectively induced cell cycle arrest on PC3 cells. In consideration of the cell cycle being regulated and controlled by a family of proteins known as cyclins and cyclin-dependent kinases (CDKs), the expression levels of cyclins and CDKs were evaluated by western blot analysis and the result was presented in Fig. 4c. Cyclin A2 is synthesized at the onset of the S phase and the cyclin A2-CDK2 complex in the nucleus is implicated in the initiation and progression of DNA synthesis [26]. Consistent with this, the expression of cyclin A2 and CDK2 was dramatically decreased in PC3 cells post treated with BP-PEI@RNAs for 24 h. Corresponding with the finding that activated cyclin B1-CDK1 promotes several early events of mitosis, such as chromosome condensation, nuclear envelope breakdown, and spindle pole assembly [27, 28], the activation of cyclin B1 and CDK1 was suppressed in PC3 cells post the treatment of BP-PEI@RNAs. Meanwhile, reduced cyclin D1 expression was detected in PC3 cells after the treatment with BP-PEI@RNAs, which was consistent with the finding that mutations, amplification and overexpression of cyclin D1 are frequently observed in a variety of tumors and may contribute to tumorigenesis [29].

### BP-PEI@RNAs treatment promoted the autophagy and apoptosis of PC3 cells

**Fig. 5:**
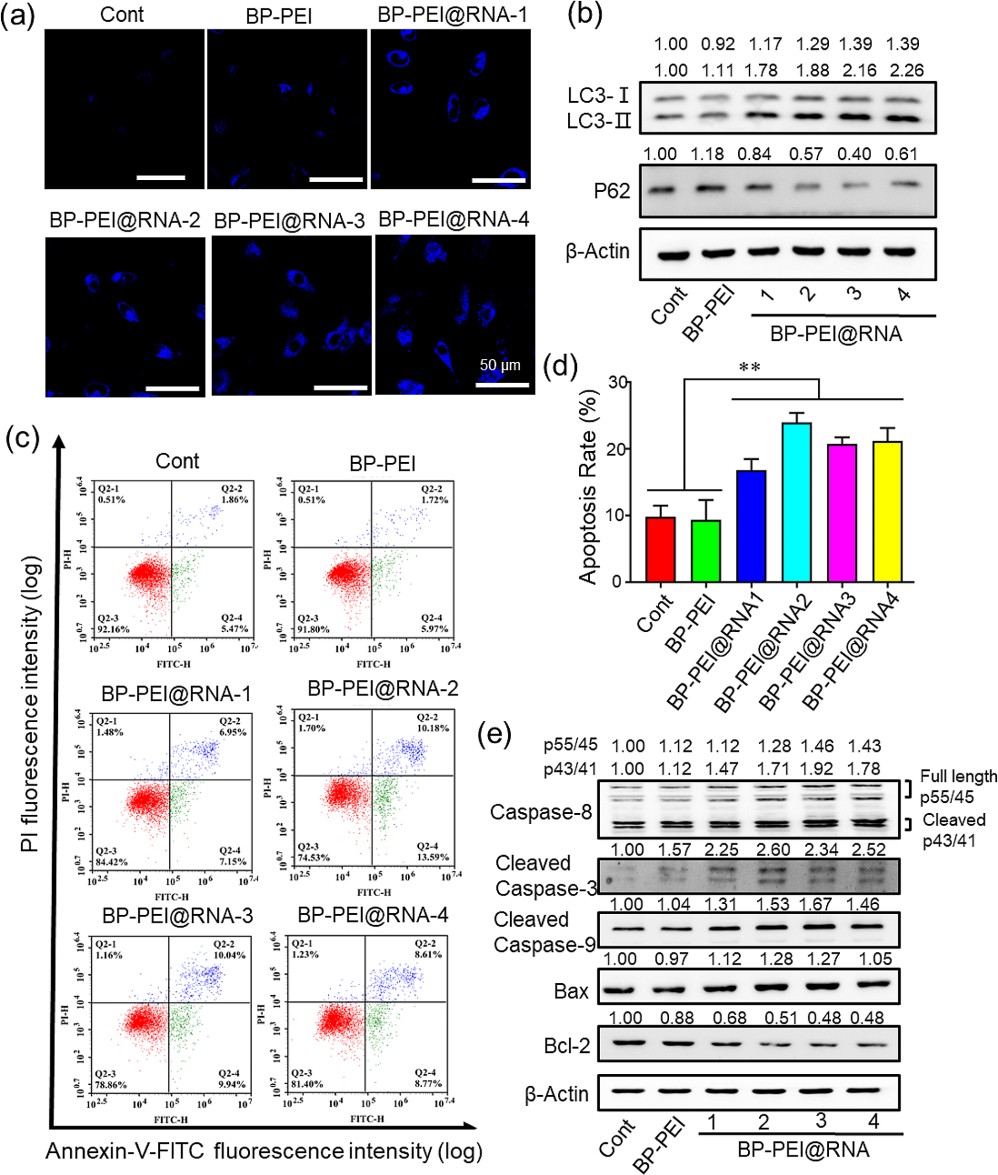
The effect of BP-PEI@RNAs on autophagy and apoptosis of PC3 cells. After PC3 cells were treated with BP-PEI@RNAs for 24 h, autophagosome in PC3 cells was stained by the Autophagy Blue™ and visualized in the DAPI channel by confocal laser microscopy (**a**), scar bar = 50 μm; The expression level of autophagy-related proteins (LC3-I, LC3-II and P62) was detected by Western-Blot assay (**b**); The apoptosis of PC3 cells was detected by flow cytometry post Annexin V-FITC/ PI staining (**c**) and the apoptosis rate was analyzed in (**d**); The expression level of apoptosis-related proteins (cleaved Caspase 8/ 3/ 9, Bax and Bcl-2) was evaluated by Western-Blot assay (**e**). ** indicated *P* < 0.01

Autophagy and apoptosis, two programmed cell death pathways, play important roles in maintaining cellular and organismal homeostasis [30]. Here, we investigated the effect of BP-PEI@RNAs treatment on autophagy and apoptosis of PC3 cells in vitro. After being treated with BP-PEI@RNAs respectively, the intracellular autophagic vesicles, which are proportional to the intensity of cellular autophagy, were stained with a fluorescent probe and observed by CLSM. As shown in Figs. 5a and S5, the number of intracellular autophagosomes (in blue) was significantly increased after BP-PEI@RNAs treatment compared with the BP-PEI group and the cont. group. This illustrated that BP-PEI@RNAs induced cellular autophagy within PC3 cells. Furthermore, the expression levels of autophagy-related proteins in PC3 cells were detected by western blot, as shown in Fig. 5b, which showed that BP-PEI@RNAs treatment led to a significant increase with the LC3-II/LC3-I ratio (which is positively correlated with the number of autophagosomes, reflecting the autophagic activity of the cells) compared with the cells in other groups, while p62 protein, degraded by lysosomal enzymes in autophagic vesicles when autophagy is activated [31, 32], was significantly decreased. Moreover, the apoptosis of PC3 cells post BP-PEI@RNAs treatment was detected by flow cytometry assay with Annexin V-FITC staining as shown in Fig. 5c-d. BP-PEI@RNAs treatment increased the apoptosis rate of PC3 cells compared with the cells in the BP-PEI group and the cont. group. However, free RNA treatment could not change the apoptosis rate of PC3 cells (Fig. S6). Furthermore, the expression levels of apoptosis-related proteins were detected by western blot assay, and the expression levels of caspase-8, caspase-3 and caspase-9 proteins (pro-apoptotic proteins) were significantly increased, among which the Bax was not very significant, while the expression levels of Bcl-2 protein (anti-apoptosis protein) were significantly decreased after BP-PEI@RNAs treatment within PC3 cells (Fig. 5e). These data demonstrated that BP-PEI@RNAs treatment could promote the autophagy and apoptosis of PC3 cells.

### BP-PEI@RNAs treatment decreased the content of mirnas within pc3 cells which targeted inhibiting the expression of PTEN

**Fig. 6:**
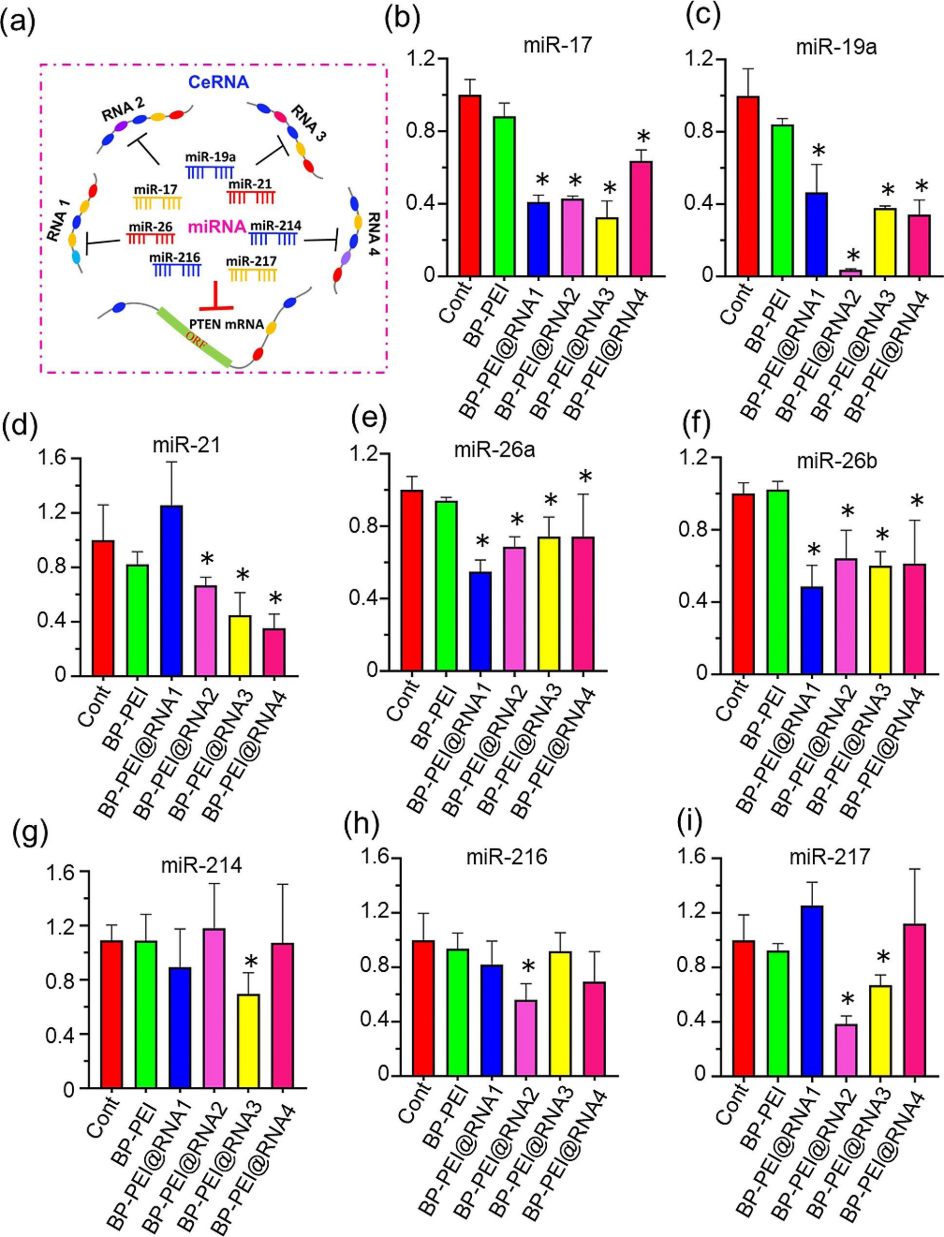
The expression levels of microRNAs target PTEN mRNA post BP-PEI@RNA treatment. (**a**) The schematic of ceRNA mechanism. (**b**)-(**i**) The expression levels of microRNAs (miR-17, miR-19a, miR-21, miR-26a, miR-26b, miR-214, miR-216 and miR-217) within PC3 cells were quantified by RT-qPCR post BP-PEI@RNAs treatment for 24 h

It was reported that long non-coding RNA PTENP1 could act as an “endogenous sponge” to share miRNA binding sites to block the degradation effect of miRNAs on the PTEN mRNA through ceRNA mechanism (the scheme was shown in Fig. 6a) [33]. In this study, we aimed to elevate the PTEN expression level by RNAs with the sequence derived from PTENP1. Therefore, to further illustrate the mechanism of how the BP-PEI@RNAs elevated the expression level of PTEN in PC3 cells, the content of several miRNAs (miR-17, miR-19a, miR-19b, miR-21, miR-26a, miR-26b, miR-214, miR-216 and miR-217) which were validated could bind PTEN mRNA and participate its degradation process in previous studies were quantified by RT-qPCR [34–37]. As shown in Fig. 6b-i, treated with all of four different BP-PEI@RNAs respectively, the content of miR-17, miR-19a, miR-26a and miR-26b within PC3 cells were significantly decreased. The expression level of miR-21 was also decreased in PC3 cells treated with BP-PEI@RNAs (except BP-PEI@RNA1). In addition, the expression levels of miR-214, miR-216 and miR-217 were downregulated within PC3 cells in BP-PEI@RNA3, BP-PEI@RNA2, BP-PEI@RNA2, 3 treatment groups, respectively. (Fig. 6g-i). These data suggested that BP-PEI@RNAs possibly reduced the degradation of PTEN mRNA in PC3 cells by sponging with miRNAs, thereby increasing the intracellular PTEN expression level and ultimately exerting tumor suppressive effects.

### BP-PEI@RNAs inhibited tumor growth in the PC3 tumor models

**Fig. 7:**
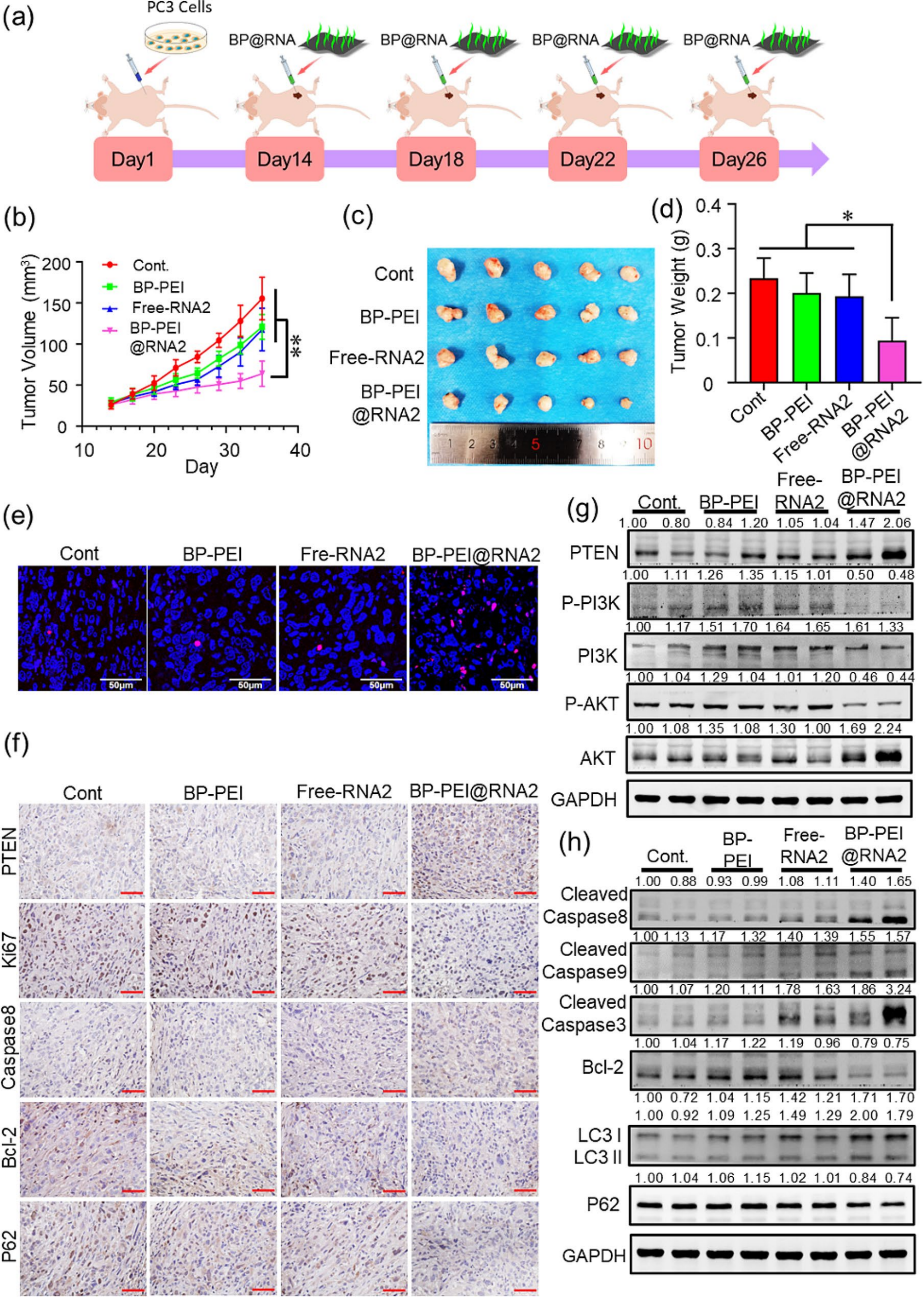
The anti-tumor effect and mechanism of BP-PEI@RNAs on prostate cancer in vivo. The schematic of the animal experiment was presented in (**a**). Briefly, male Balb/C nude mice received subcutaneous injection of PC3 cells on the first day. Two weeks later, the mice received intra-tumor injections of BP-PEI@RNA2 several times (on the 14th, 18th, 22nd, and 26th day). The volume of tumors was measured every 3 days (**b**). On the 35th day, the mice were sacrificed and tumors were harvested (**c**) for weight measurement (**d**) and histological tests. Cellular apoptosis in PC3 tumors was detected by TUNEL assay (**e**), the nucleus in blue, cells undergoing apoptosis in red, scar bar = 50 μm. The expression level of proteins (PTEN, Ki67, Caspase8, Bcl-2 and P62) in tumor tissues was determined by IHC (**f**), and the protein expression levels related to cellular proliferation (PI3K and AKT) (**g**), apoptosis (Caspase 3/ 8/ 9 and Bcl-2), and autophagy (LC3 and p62) (h) were determined by Western-Blot assay

Further, the tumor suppression effect of the BP-PEI@RNAs in vivo was determined within PC3 tumor-bearing nude mouse models, and BP-PEI@RNA2 was adopted in this section as a representative. Fourteen days post PC3 cells subcutaneous injection when the tumors grew to the size of about 50 mm^3^, BP-PEI@RNA2 was intra-tumor injected every 5 days for 4 times and the changes of the tumor volume were monitored. As shown in Fig. 7a ~ c, BP-PEI@RNA2 intra-tumor injection could effectively inhibit tumor growth when compared with the mouse models in other groups. TUNEL fluorescence staining was adopted to detect the apoptosis rate within the tumor tissues, and many more apoptotic cells (red fluorescence) were observed in the tumor tissue section which was treated with BP-PEI@RNA2 than that in the other three groups (Fig. 7e). Next, immunohistochemical staining was adopted to evaluate the expression level of PTEN protein and proteins that play a vital role in the biological process of tumor proliferation (Ki67), apoptosis (Caspase8 and Bcl2) and autophagy (P62). As shown in Fig. 7g, BP-PEI@RNA2 intra-tumor injection notably enhanced the content of PTEN protein in the tumor tissues, thus demonstrating the efficacy of BP-PEI@RNA2 in increasing the expression level of PTEN protein in vivo. Meanwhile, BP-PEI@RNA2 treatment inhibited the expression level of Ki76, Bcl2 and p62 which could promote cell proliferation, inhibit cell apoptosis and autophagy respectively, and enhanced the expression level of Caspase 8 which was involved in promoting cell apoptosis. Furthermore, western blot analysis was adopted to evaluate the changes in the levels of PTEN and its downstream cascade effector molecules which are involved in cell proliferation (PI3K and AKT), autophagy (LC3 and P62) and apoptosis (Caspase 3/ 8/ 9 and Bcl-2), and two tumor tissues were randomly selected in each group (Fig. 7f, g and h). Similar observations were found in the data in vitro, the expression level of PTEN protein was elevated and the activated form of PI3K and AKT (P-PI3K and P-AKT) were decreased post BP-PEI@RNA2 treatment. In addition, BP-PEI@RNA2 treatment facilitated the level of cleaved caspases 3/ 8/ 9 and suppressed the expression of Bcl-2. Moreover, autophagy-related protein LC3 was elevated and p62 was decreased post BP-PEI@RNA2 treatment. Thus, these data confirmed the therapeutic effect of BP-PEI@RNA on PCa by enhancing the expression level of tumor suppress gene PTEN in vivo.

## Conclusion

In summary, the sequence of long non-coding RNA PTENP1 was adopted as a template to synthesize four different RNA segments and was then loaded onto PEI-modified BP nanosheets to construct the BP-PEI@RNAs. This study revealed that BP-PEI nano-sheets could effectively transport RNAs into PC3 cells and acted as “endogenous sponges” to competitively bind miRNAs which could interact with PTEN mRNA and induce its degradation to elevate PTEN expression, and it could dramatically inhibit cell proliferation and enhance cell apoptosis, autophagy and cell cycle arrest to exert an obviously anti-tumor effect on PCa. It is a promising gene therapeutic platform for PCa treatment and provides a new perspective on tumor management.

### Electronic supplementary material

Below is the link to the electronic supplementary material.

Supplementary Material 1

## Data Availability

No datasets were generated or analysed during the current study.
