# Supplementary material for: A black phosphorus nanosheet-based RNA delivery system for prostate cancer therapy by increasing the expression level of tumor suppressor gene PTEN via CeRNA mechanism
Source: J Nanobiotechnology. 2024 Jul 4;22:391. doi: 10.1186/s12951-024-02659-2 (PMC11223337; doi:10.1186/s12951-024-02659-2)
Supplement: Supplementary file 1 — Supplementary Material 1 [file 12951_2024_2659_MOESM1_ESM.docx]

*Supporting Information*

**A Black Phosphorus Nanosheet-based RNA Delivery System for Prostate Cancer Therapy by Increasing the Expression Level of Tumor Suppressor Gene PTEN *via* CeRNA Mechanism**

Shunye Su**^†^** ^#^, Leyi Liu**^†^** ^#^, Qingfeng Fu**^†,^** ^#^, Minghao Ma**^‡^**, Na Yang^ф^, Ting Pan**^§^**, Shengyong Geng^ф^, Xue-Feng Yu^ф^, Jianqiang Zhu**^†^**^*^

**^†^** Department of Urology, Tianjin Institute of Urology, The Second Hospital of Tianjin Medical University, Tianjin 300211, China.

**^‡^** State Key Laboratory of Environmental Chemistry and Ecotoxicology, Research Center for Eco-Environmental Sciences, Chinese Academy of Sciences, Beijing 100085, China.

**^ф^**Materials and Interfaces Center, Shenzhen Institutes of Advanced Technology, Chinese Academy of Sciences, Shenzhen 518055, China.

**^§^** Institute of Nanophotonics, College of Physics & Optoelectronic Engineering, Jinan University, Guangzhou 511443, China.

* Corresponding authors: Jianqiang Zhu, Ph.D.

Tel: 86022-88329692; E-mail: [zjqwfmu@126.com](mailto:zjqwfmu@126.com)

SY. S, LY. L and QF. F. are co-first authors

**SUPPLEMENTARY FIGURE AND FIGURE LEGEND**


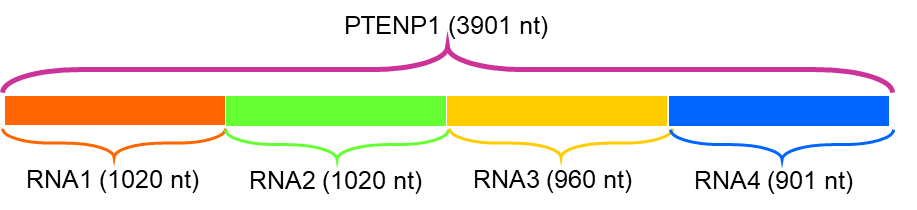


**Figure S1.** The whole length of PTENP1 (3901 nt) was randomly divided into four segments as templates to synthesize RNA1 (1020 nt), RNA2 (1020 nt), RNA3 (960 nt) and RNA4 (901 nt).


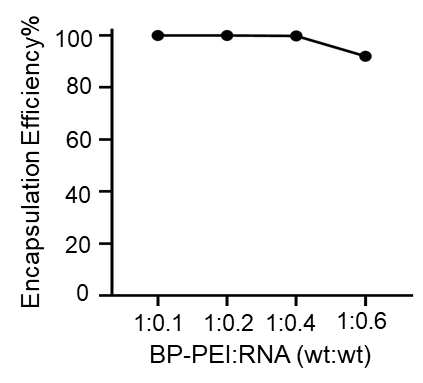


**Figure S2.** RNA encapsulation efficiency with different mass ratios of BP-PEI: RNA.


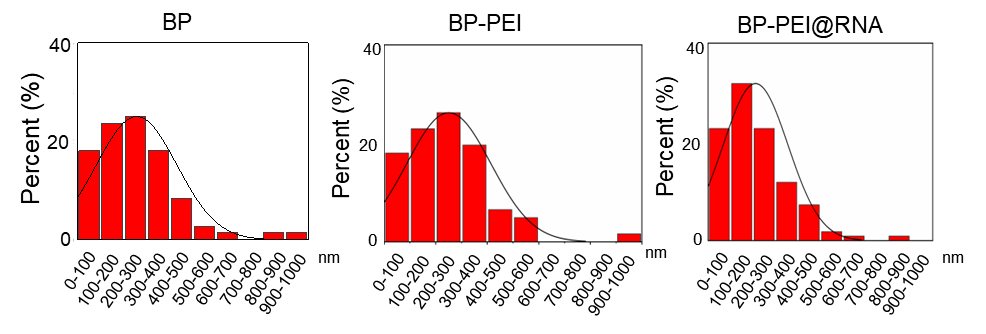


**Figure S3.** The size distribution of BP, BP-PEI and BP-PEI@RNA based on the AFM analysis.


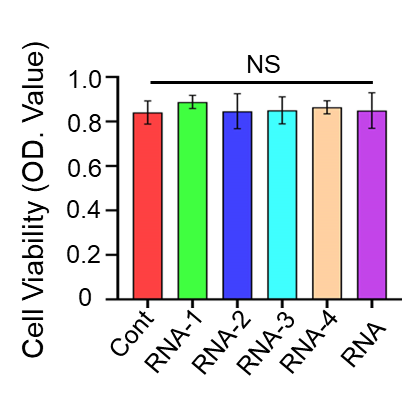


**Figure S4.** Treatment of the four segments of RNA alone or in combination had no effect on the viability of PC3 cells.


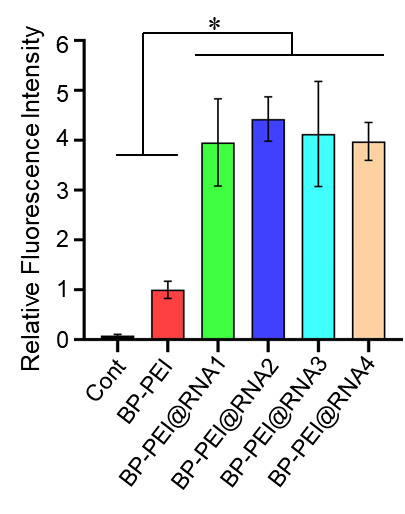


**Figure S5.** The quantitative data of the autophagosome fluorescence in PC3 cells after being treated with BP-PEI@RNAs for 24 hours and stained by Autophagy Blue™.


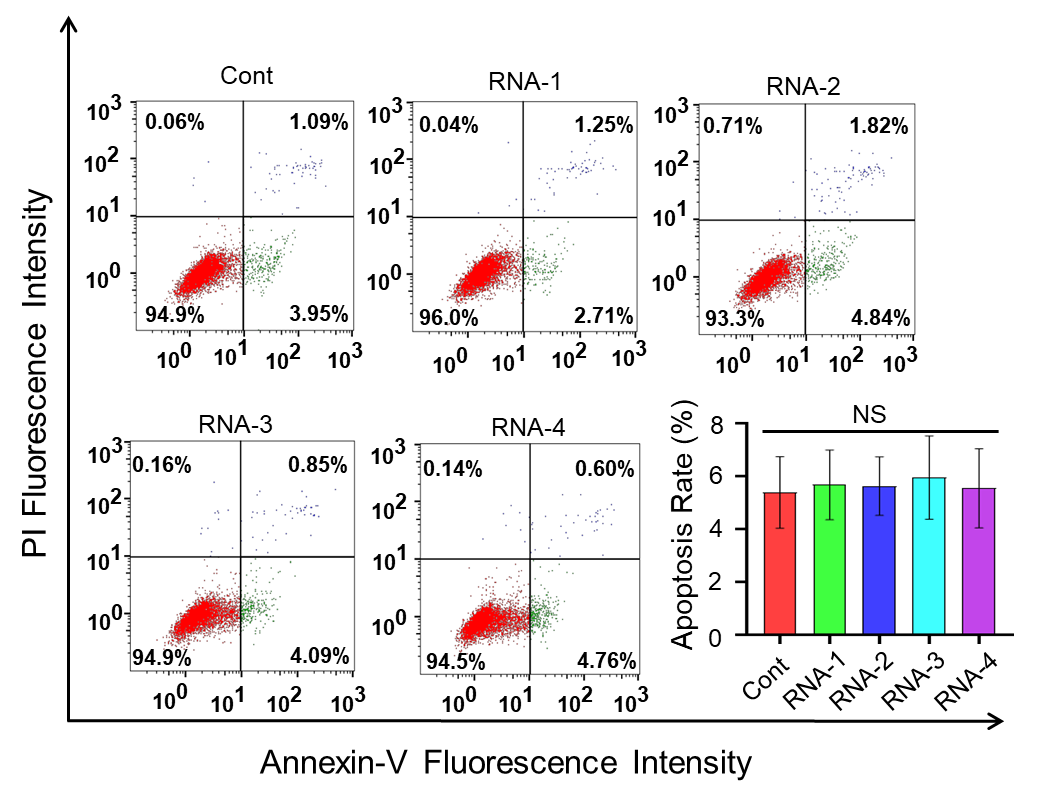


**Figure S6.** RNAs alone treatment could not promote apoptosis of PC3 cells.

**Table S1**. The primers for RNAs synthesis

| Name | Forward (5’~3’) | Reverse (5’~3’) |
| --- | --- | --- |
| RNA1 | AGATGAGAGACAGAGACGGCG | CTGTAATTAGATTTGGCGGTGTCATAATGTCT |
| RNA2 | AGTTGCGCAATATCCTTTTGAAGAC | CTTTTCAGTTTATTCAAGTTTGTTTTCATGGTGT |
| RNA3 | GGACCTTTTTTTTTAATGGCAATAGGACA | ATTCACAGCAGGTATATGATTGAAAACTAGT |
| RNA4 | GCTTCATGTGCTGCCTGCAAG | TCTTTGTGAAGTTATAAACTTTAATTTTATTTGGTAGGTATCT |

**Table S2.** The primers for MicroRNAs RT-qPCR

| Name | Forward (5’~3’) | Loop (5’~3’)/ Reverse (5’~3’) |
| --- | --- | --- |
| miR-17 | CGGCGGCAAAGTGCTTACAGTGC | GTCGTATCCAGTGCAGGGTCCGAGGTATTCGCACTGGATACGACCTACCT |
| miR-19a | CGGCGGTGTGCAAATCTATGC | GTCGTATCCAGTGCAGGGTCCGAGGTATTCGCACTGGATACGACTCAGTT |
| miR-21 | CGGCGGTAGCTTATCAGACTGATG | GTCGTATCCAGTGCAGGGTCCGAGGTATTCGCACTGGATACGACTCAACA |
| miR-26a | CGGCGGTTCAAGTAATCCAGG | GTCGTATCCAGTGCAGGGTCCGAGGTATTCGCACTGGATACGACAGCCTA |
| miR-214 | CGGCGGACAGCAGGCACAGACAG | GTCGTATCCAGTGCAGGGTCCGAGGTATTCGCACTGGATACGACACTGCC |
| miR-216 | AGCGCCTAAATCTCTGCAGGC | GTCGTATCCAGTGCAGGGTCCGAGGTATTCGCACTGGATACGACTCACAT |
| miR-217 | AGCGAGGCTACTGCATCAGGA | GTCGTATCCAGTGCAGGGTCCGAGGTATTCGCACTGGATACGACTCCAAT |
| U6 | GCTTCGGCAGCACATATACTAA | CGAATTTGCGTGTCATCCTT |
